# From bowel inflammation to the bone and joints: musculoskeletal examination in inflammatory bowel disease (IBD)

**DOI:** 10.1186/s12891-021-04903-4

**Published:** 2021-12-04

**Authors:** Samane Tavassoli, Iman Shahabinasab, Alireza Norouzi, Taghi Amiriani, Nafiseh Abdolahi, Somayeh Livani, Seyed Farzam Mirkamali, Honey Sadat Mirkarimi, Fazel Isapanah Amlashi, Sima Besharat

**Affiliations:** 1 Golestan Rheumatology Research Center, Golestan University of Medical Sciences, Gorgan, Iran; 2 Golestan Research Center of Gastroenterology and Hepatology, GolestanUniversity of Medical Sciences, 3rd floor, Heart Complex, Sayyad-e-Shirazi Hospital, Sayyad-e-Shirazi Boulevard, Gorgan city, Golestan province Iran; 3 Clinical Research Development Unit (CRDU), Sayad Shirazi Hospital, Golestan University of Medical Sciences, Gorgan, Iran

**Keywords:** Inflammatory bowel disease, Sacroiliitis, Enthesitis

## Abstract

**Background:**

One of the most important complications in inflammatory Bowel Disease (IBD) are musculoskeletal manifestations that are reported in more than 50% of patients.

**Objectives:**

In this study, we aimed to evaluate the musculoskeletal and radiologic manifestations in our IBD patients.

**Methods:**

In this cross-sectional study on 96 mild-to-moderate IBD patients (76 UC, 18 CD and 2 undifferentiated IBD) with mean (SD) age of 39.28 (11.42) years, 44 (45.8%) were males and 52 were (54.2%) females. Patients were examined by an expert rheumatologist and their musculoskeletal symptoms were assessed. The musculoskeletal system was evaluated by Modified Schober test, Thoracic expansion (TE), Occiput to wall distance (OWD), and Patrick’s or FABER test. Peripheral joints were also examined in all four extremities. Then patients were referred for pelvic and lumbosacral x-ray. Sacroiliitis grading was performed using the New York criteria.

**Results:**

Inflammatory low back pain was reported in 5 (5.2%), enthesopathy in 6 (6.5%) and dactylitis in 1 (1.1%). Positive Schober test was recorded in 5 (5.2%) and Patrick test in 3 (3.1%). Forty-nine (51%) cases had normal imaging with no sacroiliitis, endplate sclerosis was seen in 33 cases (34.4%), grade 3 and grade 4 were seen in 10 cases (10.4%).

**Conclusions:**

In the present study, 34.4% of the IBD patients had mild radiologic changes as endplate sclerosis and 95% had a normal physical examination.

## Background

Inflammatory bowel disease (IBD) is a chronic inflammatory disease composed of Crohn’s disease (CD) and ulcerative colitis. The etiology of IBD remains unknown but it is believed that the interaction of genetics, environment and immune system play a major role [1]. There is no curative therapy for IBD and the goal of treatment is to prevent complications and reducing the progression of inflammation [2].

Extra intestinal manifestations (EIM) are common in IBD patients. More than half of the patients experience at least one extra intestinal symptom during their lifetime [3]. The incidence of EIM varies from 6 to 47%. Extraintestinal manifestations of IBD can affect many systems in the body such as musculoskeletal, ocular, dermatologic, hepatobiliary and etc. But one of the most common complications of IBD is musculoskeletal (MSK) manifestations. Approximately more than 50% of IBD patients, develop MSK complications including axial and peripheral arthritis [4–6].

Axial arthritis consists of sacroiliitis and ankylosing spondylitis (AS), which are not associated with intestinal disease activity. Ankylosing Spondylitis has been reported in 5 to 10% of IBD patients. These patients complain about back pain and dryness at night, during the morning, and after immobility. Pure sacroiliitis is a common finding (up to 20% of patients) but it is asymptomatic in many patients [7, 8].

Spondyloarthritis (SpA) is divided into two types: peripheral and axial. Peripheral involvement can be divided into two subtypes: subtype 1 (non-destructive) asymmetrically affects the large joints including the knee, hip, wrist, elbow, and ankle that associated with bowel disease activity. Mostly it lasts only a few weeks with no clear radiologic manifestations. But subtype 2 (destructive) involves the small joints symmetrically and has no clear association with IBD [9–11].

Common available medications for IBD are Anti-TNF alpha drugs, amino salicylates, corticosteroids and immunomodulators. Corticosteroids have systemic effects and maybe there will be limits in some patients in order to use them. Recent studies have shown that Anti-TNF agents are effective to induce remission in both adults and children. Infliximab and Adalimumab are the only Anti-TNF agents that approved by Food and drug administration (FDA). Prescribing these drugs has reduced the use of corticosteroids [12].

The treatment of IBD and rheumatologic musculoskeletal complications are similar to the treatment of IBD itself (using 5-ASA combinations like Sulfasalazine), but the question is, does IBD treatment affects MSK? [13, 14].

In this study, we aimed to evaluate the musculoskeletal and radiologic manifestations in IBD patients.

## Method

### Study population and design

In this cross-sectional study, 100 registered IBD patients were recruited: 4 were excluded during the study because of pregnancy, among 96 remained patients, 76 were UC, 18 Crohn’s disease and 2 undifferentiated IBD. Patients were invited to the Golestan Research Center of Gastroenterology and Hepatology (GRCGH) by telephone call.

#### Inclusion criteria

All IBD patients registered in the IBD bank have been reached out through the telephone, and recruited into the study if agreed to terms of the study.

#### Exclusion criteria

Hospital admission at the time of study and during the last month, history of fracture or trauma after the diagnosis of IBD, pregnancy and not willing to have an x-ray were among the exclusion criteria.

Patients were examined by an expert rheumatologist and their musculoskeletal symptoms were assessed throughout the following tests to evaluate the musculoskeletal symptoms: modified Schober test, Thoracic expansion (TE), Occiput to wall distance (OWD) and Patrick’s test or FABER test [15]. Peripheral joints of all four extremities were also examined.

### Radiological evaluation

After finishing the physical examination and completing the questionnaire throughout a face-to-face interview, patients were referred to a well-equipped imaging center to perform a pelvic and lumbosacral x-ray. The radiologist blinded to the rheumatologic exam findings.

Sacroiliitis grading was performed using the New York criteria [16]:

- Grade 0: Normal imaging
- Grade 1: some blurring of the joint margins (Suspicious)
- Grade 2: Minimal sclerosis with some erosion
- Grade 3: definite sclerosis on both sides of joint / severe erosions with widening of joint space with or without ankyloses
- Grade 4: complete ankyloses

Radiologic reports were all seen and graded by one expert radiologist. Those patients with problems in their X-ray were referred for further managements. Rheumatologic and radiologic findings analyzed by another rheumatologist.

## Results

In this study on 96 IBD patients (76 UC, 18 CD and 2 undifferentiated IBD) with mean (SD) of 39.28 (11.42) years, there were 44 (45.8%) males and 52 (54.2%) females. Table 1 shows the demographic variables of the study population.

**Table 1:** Demographic and anthropometric data of the studied population of IBD

Age, Mean (SD), years	39.28 (11.42)
Sex, N (%)	
Male	44 (45.8)
Female	52 (54.2)
Type of IBD, N (%)	
UC	76 (79.2)
CD	18 (18.8)
Undifferentiated	2 (2.1)
Duration of the bowel disease, Median (SE)	5 (0.65)
Body Mass Index (BMI), Mean (SD), kg/m2	26.26 (4.36)
BMI group, N (%)	
Underweight (< 18.5)	1 (1)
Normal (18.5–24.9)	52 (54.2)
Overweight (25–29)	27 (28.1)
Obese (≥30)	16 (16.7)
Waist circumference, Mean (SD), cm	89.35 (10.40)
Abdominal circumference, Mean (SD), cm	97.4 (10.97)
Medication, N (%)	
Sulfunamides (Asacol, Mesalazine, Sulfasalazine)	78 (81.2)
Anti-TNF (Remicade, Cinnora)	18 (18.8)
Azathioprine	39 (40.6)
Prednisolone	37 (38.5)

History taking and physical examination showed inflammatory low back pain in 5 (5.2%), enthesopathy in 6 (6.5%) and dactilitis in 1 (1.1%). Rheumatologic examinations of the studied population showed positive Schober test in 5 (5.2%) and positive Patrick test in 3 (3.1%) Table 2.

**Table 2:** Results of the rheumatologic examinations in IBD patients

Occiput to Wall Distance, Mean (SD), cm	4.31 (1.66)
Schober test, N (%)	
Positive	5 (5.2)
Negative	91 (94.8)
Schober index, Mean (SD), cm	6.94 (1.30)
Patrick test, N (%)	
Positive	3 (3.1)
Negative	93 (96.9)
Inflammatory Low Back Pain, N (%)	
Positive	5 (5.2)
Negative	91 (94.8)
Peripheral arthropathy, N (%)	
Upper extremities	
Left	2 (2.1)
Right	2 (2.1)
Lower extremities	
Left	2 (2.1)
Right	2 (2.1)
Dactilitis, N (%)	1 (1.1)
Enthesopathy, N (%)	6 (6.5)

Lumbosacral and pelvic X-ray reports are shown in Table 3. Forty-nine (51%) cases had normal imaging with no sacroilleitis, endplate sclerosis was seen in 33 cases (34.4%), and definite sclerosis on both sides with or without ankyloses (grade 3) and complete ankyloses (grade 4) were seen in 10 cases (10.4%).

**Table 3:** Radiologic manifestation in IBD patients

End plate sclerosis in Lumbosacral joint, N (%)	33 (34.4)
Sacroilleitis grades, N (%)	
Grade 0 (Normal imaging)	49 (51)
Grade 1 (Suspicious)	14 (14.6)
Grade 2 (Minimal sclerosis with some erosion)	23 (24)
Grade 3 (definite sclerosis on both sides with or without ankyloses)	8 (8.3)
Grade 4 (complete ankyloses)	2 (2.1)

Twenty one patients were taking Anti-TNF drug (22%), 42 (44%) Azathioprine and 40 (42%) were taking Prednisolone. Because of small number of patients who were taking Anti-TNF medication, it is not possible to find significant relationship between treatment and radiologic manifestations. Only 12% of patients who were treating by Anti-TNF medications had grade 2 or higher sacroiliitis. (Vs. 24.6% in patients who were not taking Anti-TNF).

## Discussion

In this study regards to the evaluation of the musculoskeletal manifestations in patients with IBD, severity of the disease was measured on the basis of New York criteria and musculoskeletal symptoms were assessed on the basis of radiological observations and physical examination of patients. Numerous reports from different countries showed various range of rheumatologic symptoms (2 to 46%) [17].

In our study, patients had few obvious musculoskeletal symptoms as inflammatory low back pain in 5.2%, enthesopathy in 6.5% and dactylitis in 1.1%. This may be due to the treatment with immunosuppressive and immunomodulatory medications [18]. As mentioned before, approximately 50% of IBD patients experience at least one rheumatologic manifestation in their lifetime, but the mean duration of the disease was 5 years in the present study. During the years after the first diagnosis, the probability of rheumatologic manifestations would be more prominent. So, some may develop rheumatologic complications in the next coming years.

The small number of patients compared to the studies mentioned can be a reason for minor differences in radiological results as well as physical examinations. Interestingly, the study by Giani et al. performed on 34 IBD patients, no one had symptoms in physical examination, but sacroiliitis reported in 15% of them on Magnetic Resonance Enterography (MRE) [19].

In our study, 34.4% of patients had radiologic changes as end plate sclerosis, but positive physical exams were seen in less than 5% of them. The bowel activity index was more than 6 just in 4 cases indicating that most of our patients were in the remission phase.

The modality used for evaluation of sacroiliitis can affect the result. As in a study in Canada the prevalence of sacroiliitis was three times higher in IBD patients than in the control group evaluated by CT scan [20].

In this regards, a review study in Italy stated that pelvic radiography often identifies SpA in its late stages and MRI is the goal standard diagnostic imaging for assess SpA [21].

Another study in France showed a 9.8% prevalence of sacroiliitis in their IBD patients with CT scan and 15.7% with MRE [22].

But another study in Canada found sacroiliitis in 16% of their IBD patients through radiological examinations [23], similar to our results.

A study in Italy has also reported that some patients with asymptomatic IBD have radiologic evidence of spondyloarthritis [6]. Asymptomatic patients are often less treated and less likely to adhere to treatment than symptomatic patients. On the other hand, asymptomatic patients have fewer referrals to a physician, and their rheumatologic symptoms are expected to be diagnosed later.

As shown in another study from Korea, ankylosing spondylitis and rheumatoid arthritis are more common in IBD patients than other rheumatologic diseases [24].

In the present study, endplate sclerosis was seen in 34.4% and higher grade of sacroilliitis (grade 3 and 4) was reported in 10.4% of IBD cases, although they were clinically asymptomatic, probably due to the prescribed treatment.

It has been suggested that genetic factors, and even the microbiome composition of IBD patients, could make a difference in musculoskeletal manifestations [25]. Therefore, the potential role of genetics in the presence of extra intestinal symptoms in patients should be considered. Further studies are needed to investigate these factors.

## Conclusion

In our study, one-third of patients (34.4%) had mild radiologic changes as endplate sclerosis and 94–95% of patients had a normal physical examination. Therefore, it can be concluded that even patients who have radiologic manifestations may have normal physical examinations. Medication use and short duration of illness are probably important reasons for the normal physical examination of our patients.

### Limitations

One of the most important limitation of this study was the small number of patients. And the other one was a Poor patient cooperation due to difficulty in referral or dissatisfaction. So, we were unable to follow them to assess whether symptoms changes or not.

## Data Availability

The datasets used and analyzed during the current study but not publicly available due to limitations of ethical approval involving the patient data and anonymity but are available from the corresponding author on reasonable request.
